# Predictors of Posttraumatic Stress and Posttraumatic Growth in Childhood Cancer Survivors

**DOI:** 10.3390/cancers9030026

**Published:** 2017-03-16

**Authors:** Veronika Koutná, Martin Jelínek, Marek Blatný, Tomáš Kepák

**Affiliations:** 1 Institute of Psychology of the Czech Academy of Sciences, Brno, 60200, Czech Republic; jelinek@psu.cas.cz (M.J.); blatny@psu.cas.cz (M.B.); 2 Department of Pediatric Oncology, Children’s Medical Center, The University Hospital Brno, Brno, 60200, Czech Republic; Kepak.Tomas@fnbrno.cz

**Keywords:** posttraumatic stress, posttraumatic growth, benefit finding, childhood cancer survivors

## Abstract

This longitudinal study aims to analyze predictors of posttraumatic stress symptoms (PTSS) and posttraumatic growth (PTG) among gender, age, objective factors of the disease and its treatment, family environment factors and negative emotionality. The sample consisted of 97 childhood cancer survivors (50 girls and 47 boys) aged 11–25 years who were in remission 1.7 to seven years at T1 and four to 12.5 years at T2. Survivors completed a set of questionnaires including the Benefit Finding Scale for Children and the University of California at Los Angeles Posttraumatic Stress Disorder Index. Regression and correlation analyses were performed. The relation between PTSS and PTG was not proven. A higher level of PTSS (T2) was associated with higher levels of negative emotionality (T1). A higher level of PTG (T2) was connected to a higher level of warmth in parenting (T1), female gender and older age at assessment. Medical variables such as the severity of late effects and the time from treatment completion did not play a significant role in the prediction of PTSS and PTG. PTG and PTSS are more influenced by factors of parenting and emotional well-being of childhood cancer survivors than by objective medical data.

## 1. Introduction

Oncologic disease and its treatment in childhood represent a specific form of traumatic event [1]. Posttraumatic stress symptoms (PTSS) and posttraumatic stress disorder (PTSD) in terms of symptoms of re-experiencing, avoidance/numbing and increased arousal have been widely studied in childhood cancer survivors. According to the review by Taïeb et al. [2], various studies have found 2%–20% of childhood cancer survivors report symptoms consistent with PTSD. However, current research shows most children and adolescents adapt to this situation quite well and the prevalence of psychopathological problems including PTSS/PTSD is often reported as identical with the general population or even lower [3,4,5,6]. Studies focused on the monitoring of adverse psychosocial consequences of childhood cancer were recently extended with a focus on the possible positive effects of this experience. Not only do childhood cancer survivors usually adapt to this situation quite well, but according to Barakat et al. [7], more than 80% of adolescent survivors of childhood cancer report at least one positive consequence of this experience, referred to in literature as posttraumatic growth [8].

### 1.1. Factors Influencing PTSS/PTSD and Posttraumatic Growth (PTG)

The incidence of PTSS in childhood cancer survivors has been related to belonging to the female gender, subjective factors associated with the disease and its severity assessment (e.g., perceived level of threat to life and intensity of treatment), shorter time interval after the end of treatment, poor social support and problematic family relationships, quantity and severity of physical late effects, degree of anxiety and social support upset [2,9,10,11]. PTSD incidence among childhood cancer survivors may also depend on age. Stuber et al. reported a higher prevalence of PTSD for young adult survivors than for child and adolescent survivors and an increased risk of PTSD associated mainly with intensive treatment, lower education, being unmarried, lower income and unemployment [12].

The occurrence of PTG among childhood cancer survivors has been associated with a higher age at diagnosis and shorter periods after diagnosis or treatment completion [7,13]. Michel et al. reported higher levels of PTG in children who are convinced that the disease is still affecting their lives than in those who consider the disease to be over [14]. Some studies reported differences in the rates of PTG among sexes, but most did not find significant gender differences [15].

In the adult population, cognitive processes play the key role in the processing of traumatic events. These processes assume a certain level of cognitive maturity. The minimal age necessary for such processing is derived from two developmental milestones. Children under five years of age at diagnosis have difficulties in understanding or recalling their illness and its treatment, and they report fewer PTSS and fewer positive outcomes [7]. The ability to experience opposing (positive and negative) emotions to the same subject develops at 11 years of age [16]. The ability of introspection also falls approximately in this period. Although studies in adults mention a negative relationship between PTG and age [17], in adolescents, the relationship between PTG and age is the opposite and older adolescents report positive changes more frequently than younger children [18]. Thus the optimal age for the occurrence of PTG appears to be late adolescence to young/middle adulthood.

The parent-child relationship may serve as a protective factor influencing the child’s response to the traumatic event, whether in terms of PTSS or PTG, and successful adaptation of children at risk is connected to parenting characterized by warmth, structure and reasonably high expectations [19]. Characteristics of the family environment relevant to PTSD incidence in childhood cancer survivors can be described in terms of cohesion and expressing or processing of emotions [20] and the degree of organization in family functioning [21]. Although research concerning specific characteristics of family environment related to PTG in childhood cancer survivors is scant, social context and the relationship with parents/caregivers are the key elements of the PTG model in children [22]. Howard Sharp et al. [23] described youth’s perceptions of parental reactions as possible mechanisms facilitating PTG in youth with cancer. In their study, youth with cancer who self-identified the cancer-related event as the most stressful life event (approximately half of the sample) perceived higher levels of support and reassurance/distraction (diminishing of distress with reassurance and distraction) in their parents’ reaction and reported greater PTG than those referring to a non-cancer event.

### 1.2. Relation between PTS and PTG

In the view of Tedeschi and Calhoun [8], PTG and PTSS do not constitute two poles of a continuum, but rather two separate dimensions. The presence of PTG does not necessarily indicate a decrease of distress. Not only may PTSS and PTG coexist, but according to these authors, PTG appears as an epiphenomenon of the effort to cope with adverse circumstances that cause a high degree of psychological stress. However, the relation of PTSD and PTG has not been clearly proved yet, and four different options can be considered [24]. The first one states that this relationship is positive because PTG can occur only in the presence of PTSS [8]. The second argues that PTSS undermines the functioning and quality of life and thus prevents PTG; the relationship is therefore negative [25]. According to the third option, the curve showing the relationship between these two variables has the form of an inverted “U” [24]. This theory assumes that there is an optimal level of PTSS at which PTG occurs, and the highest PTG occurs at the middle level of PTSS. The fourth view considers PTSS and PTG to be independent of each other [26]. Tillery et al. [27] suggest that the relationship of PTSS and PTG depends on the context of the examination and that PTG within the specific context of cancer is more likely to be experienced without PTSS.

### 1.3. PTSS/PTG and Overall Adaptation to Childhood Cancer

Research suggests possible connections between PTG and the overall adaptation of children and adolescents to traumatic events. According to available studies, a positive relationship between PTG and self-esteem [13] and optimism [13,28], and a negative relationship with depressive symptoms [29] can be considered. PTG strongly correlates with positive affect, but not with negative affect, suggesting that PTG reflects a specific form of positive adaptation to a traumatic event [28].

According to Diagnostic and Statistical Manual of Mental Disorders (5th edition, DSM-5) [30], the diagnosis of PTSD is likely associated with one other mental disorder (e.g., depressive and anxiety disorders). In young adult survivors of pediatric cancer, PTSD is associated with higher levels of psychological distress and lower quality of life [31]. Young adult survivors of childhood cancer with PTSD score higher in all subscales of the Brief Symptom Inventory, a measure assessing symptoms of psychopathology including somatization, interpersonal sensitivity, depression, anxiety, obsessive-compulsive, hostility and a phobic anxiety subscale [32].

### 1.4. Current Study

This study is a part of Quality of Life Longitudinal Study in Pediatric Oncology Patients (QOLOP)—a prospective longitudinal study initiated in the year 2006 dealing with the study of the quality of life of childhood cancer survivors at the Clinic of Pediatric Oncology in Brno. The principal aim of the project is to identify impaired areas of quality of life in survivors, both in terms of objective parameters and subjective perception of contentment.

Although factors contributing to the risk of PTSS/PTSD in childhood cancer survivors are quite well documented, little is known about factors facilitating posttraumatic growth in this population and the role of parents in this process [23]. We proceed from the assumption that PTG cannot be fully comprehended unless appropriate attention is devoted to the study of the distress which preceded it, and that the process of recovering from PTSS can hardly be fully comprehended unless the possibility of PTG is taken into account; therefore, we adopt a complex perspective, integrating knowledge about PTSS as well as PTG.

This longitudinal study aims to find predictors of both PTSS and PTG among demographic variables (age and gender), objective factors of the disease and its treatment (severity of late effects, time since treatment completion), family environment factors (inconsistency, involvement, control and warmth in parenting) and negative emotionality. In this study we focus primarily on PTG in terms of benefit finding, defined as the perception of positive changes as a result of a traumatic event that may or may not be true [33]. Considering the age range of our sample and the procedure of assessment, we are interested in the subjective perception of benefits in very specific aspects of life rather than appraisal of real and persistent changes in relatively complex and more abstract domains.

## 2. Results

### 2.1. Sample and Procedure

The sample reported in this paper is a subset of a larger group of children and adolescents enrolled in the QOLOP project. Respondents were recruited from the Clinic of Pediatric Oncology in Brno, one of the two major centers for the treatment of pediatric cancer in the Czech Republic. The response rate at T1 of the QOLOP project was 96.7%.

A total of 120 childhood cancer survivors completed the T1 and T2 stages, but 23 of them were administered only a shortened version of the method set in T1 (including only the Minneapolis-Manchester Quality of Life questionnaire (MMQL)instead of the entire method set) due to attention deficit and hyperactivity issues (three respondents), cognitive deficits (two respondents), fatigue (five respondents) and insufficient maturity (13 respondents) and therefore were not included in longitudinal analyses. Further details about the whole sample and the sample with available data for longitudinal analyses can be found in Table 1.

A condition of enrollment in the first stage (T1) was the diagnosis of cancer in childhood (before the age of 18) and completion of cancer treatment, radiotherapy or chemotherapy. At T1, respondents were 7.4–19.6 (median = 10.7, mean = 11.7) years old and 1.7 to 7.0 years (median = 3.0 years; mean = 3.5 years) from the completion of successful treatment. The condition of participation in the second phase of the research (T2) was ongoing remission. The second stage (T2) was held 2.0–8.0 years after T1 (median = 4.2 years; mean = 4.5 years). The time from the completion of treatment at T2 ranged from 4.0 to 12.5 years (median = 7.7 years; mean = 8.0). Adolescents and their parents were thoroughly familiar with the course and the purpose of the study and signed an informed consent approved by the Ethics Committee of the University Hospital Brno.

Children under the age of 12 at T1 filled out the questionnaires with the assistance of a psychologist who read and explained the items (when necessary); older children completed the questionnaire alone. At T2, all of the children were older than 11 years and completed the questionnaire alone. Given that complete data from all methods were not available for some respondents, the individual analyses are supplemented with relevant information about the size of the analyzed sample. Using a 38 cut-off score for detecting PTSD by the University of California at Los Angeles Posttraumatic Stress Disorder (UCLA_PTSD) Index [34], we identified six (5%) respondents as reporting significant PTSD symptomatology.

### 2.2. Correlates of PTSS and PTG

Following a standard forward and back translation procedure, all methods used in this study were administered to respondents in the Czech language. Details about the reliability of these measures in the Czech language version can be found in the Methods section. Correlation analysis of the relationship of PTSS and PTG with each other and with other psychological characteristics reflecting the overall level of psychosocial adaptation was performed in order to further explore the validity of measures of PTSS and PTG. Based on the findings of previous studies discussed in the Introduction, we verified the relationship of PTSS and PTG with self-esteem, negative emotionality, depressive symptoms, severity of late effects and age at diagnosis. This part of the study based solely on data from T2 included 120 adolescents aged 11.5–25.0 years (median = 15.9; mean = 16.2) at T2, who completed both the T1 and T2 questionnaire set. Details about sample characteristics can be found in Table 1 (whole sample).

PTSS correlates positively with negative emotionality and depressive symptomatology and negatively with self-esteem. PTG in terms of benefit finding correlates positively with self-esteem and age at cancer diagnosis, and negatively with depressive symptomatology. The relation between PTSS and PTG and the relation of these variables to the severity of the late effects of cancer have not been proven. A summary of the results of the correlation analysis can be found in Table 2.

### 2.3. Predictors of PTSS and PTG

Only the respondents who completed the entire array of the method set at T1 provided data required for the longitudinal analysis of predictors of PTSS and PTG. Therefore, this part of the study included only 97 adolescents aged 11.5–25.0 years (median = 17.0; mean = 16.8). Further details about the sample can be found in Table 1 (sample for longitudinal analyses). To analyze predictors of PTSS and PTG, we used linear regression analysis.

Both PTSS and PTG can be predicted using the predictors of gender, severity of late effects, factors of parenting, negative emotionality and time off-treatment. These predictors explained 29% of the variance of the variables reflecting the rate of PTG and 23% of the variance of PTSS (see Table 3). Among individual predictors in the case of PTG, gender, age and parental warmth reached the level of statistical significance. A higher level of PTG (T2) is associated with female gender, older age and a warmer style of parenting (T1). In the case of PTSS, only negative emotionality reached the level of statistical significance. Survivors with higher levels of negative emotionality at T1 reported higher levels of PTSS at T2. Influence of any other considered predictors was not demonstrated by the regression analysis (see Table 3).

## 3. Discussion

The results of the correlation part of the study confirmed expected relationships of PTSS and PTG with other variables reflecting overall psychosocial adaptation. As assumed, a relation between PTG and the child’s age at cancer diagnosis was supported and the strength of this correlation is comparable to the value given by the authors of the BFSC questionnaire (r = 0.25) [13]. The relation between PTG and the age at cancer diagnosis is consistent with the theoretical assumptions about the need for certain cognitive maturity to process traumatic events, according to which PTG occurs in older children and adolescents to a greater extent/more frequently than in younger children. This result was further supported by regression analysis, where higher levels of PTG were connected to older age at the T2 assessment. However, Tremolada et al. found an opposite relation between PTG and the age at diagnosis, with younger children reporting more PTG [35]. PTG further correlates with self-esteem and depressive symptoms and PTSS is associated with lower self-esteem and higher levels of negative emotionality and depressive symptoms, which corresponds to prior studies [13,29,30,31,32].

Significant correlations of PTSS with self-esteem, negative emotionality and depressive symptomatology are stronger than significant correlations for PTG (self-esteem and depressive symptoms). The interpretation of these results may be related to the methods used for measuring individual variables. Items of the method used to measure PTSS are focused on emotions associated with the traumatic event, while items measuring PTG refer to the consequences and impact of the trauma. Methods used for the monitoring of self-esteem, depressive symptomatology and negative emotionality are also focused largely on emotions. A stronger correlation between PTSS and these variables may therefore be affected by the wording of the items.

Neither PTG nor PTSS correlate with the severity of late effects assessed by physicians. Although some studies have proven the relation between severity/number of late effects and PTSS/PTSD [36], it has been mentioned that subjective assessment and the experience of the patient are more important for the development of PTSS/PTSD than objective assessment of the state of health by physicians [2]. The results of the study of predictors of PTSS are in accordance with this finding.

The relationship of PTSS and PTG has not been supported and this result does not support the concept of Tedeschi and Calhoun [8] that PTSS is necessary for the presence of PTG. The extensive longitudinal Childhood Cancer Survivor Study came to the conclusion that the PTG and the PTSS are independent of each other [26]. Although its results show a slightly positive correlation (r = 0.11), according to the authors, its significance is caused by a very large number of respondents. According to Tillery et al. [27], PTG within the specific context of cancer is more likely to be experienced without PTSS. Our results are consistent with this assertion, but the possibility of a nonlinear relation, proposed, for example, by Levine et al. [24], in which PTG is the highest at intermediate PTSS, cannot be ruled out either. No correlation of PTSS and PTG could also be influenced by the measure of PTG used in this study. Although the Benefit Finding Scale for Children has been confirmed as a reliable instrument for assessing positive outcomes after cancer [14], it may reflect a distinct form or specific part of PTG as viewed by Tedeschi and Calhoun [8].

In the study of predictors of PTSS/PTG, we focused on the prediction of PTG and PTSS using regression analysis with predictors of gender, severity of late effects of treatment, aspects of parenting, negative emotionality and time since treatment completion. Both PTG and PTSS can be predicted based on the variables, and the overall models are significant. In the case of PTG, only parental warmth, gender and age at assessment apply from the individual variables. These results are consistent with previous studies, according to which the warmth of parental care contributes significantly to the successful adaptation and resilience of children to adverse events [37]. Characteristics of the parent-child relationships are one of the basic components of the model of posttraumatic growth in children [22] and Howard Sharp et al. [23] described youth’s perceptions of parental reactions as possible mechanisms facilitating PTG in the specific context of childhood cancer survivors. The bond between parental warmth and PTG in their child could be also related to the possibility that parents’ PTSD symptomatology might influence their child’s wellbeing. This assumption needs to be further explored.

According to the research of PTG in children following natural disasters, it appears that the parents’ ability to cope with the traumatic event that affects the child’s life and their own lives, so that they are able to provide their children with a safe haven and support, contributes to PTG in their children [38]. However, in Kilmer and Gil-Rivas’ [39] study of PTG after a hurricane, caregiver warmth was associated with the coping competency of children, but not with PTG. In their study, PTG was assessed in children seven to 10 years old at one and two years following the hurricane. Given the specifics of cancer as a traumatic event and especially its chronicity and internal nature, the influence of parental warmth may depend on the age of the children and time elapsed since trauma. Parental warmth may serve as a buffer, reducing distress and thus limiting PTG in the case of the simple and discrete nature of trauma at a younger age and with shorter time elapsed. However, the role of parental warmth may change with the growing complexity and chronicity of trauma, older age and longer time elapsed. Perhaps the reduction of distress may be substituted with the support of coping strategies leading to successful processing of traumatic event and to PTG.

In the present study, females reported higher levels of PTG than males. Although the results of the preexisting research focusing on gender differences in PTG in children and adolescents are mixed [15], Yaskowich [40] in her study of childhood cancer survivors also reported higher levels of PTG for females. Furthermore, these results are consistent with findings of a meta-analysis of PTG in youth and adults, with women reporting more PTG than men [41]. Moderator analyses conducted by Vishnevsky et al. [41] revealed that gender differences in PTG are moderated by age—with higher-mean-age women reporting incrementally more PTG than men.

From the individual variables included in the regression analysis for the prediction of PTSS, only negative emotionality applies. Negative emotional states such as fear and feelings of sadness, loneliness and inferiority may accompany PTSS, which has been corroborated by the results of the correlation part of this study. The DSM-5 [30] adds negative cognitions and mood as the fourth cluster of PTSD symptoms, in addition to the original three, distinguished in the DSM-IV (reexperiencing, avoidance, arousal). Negative emotionality may therefore rather be interpreted as a component of PTSS/PTSD than their independent predictor.

Unlike PTG, gender differences in PTSS are quite well documented in the literature, with females reporting higher levels than males [9]. However, in this study, the influence of gender has not been demonstrated. An explanation for this finding may be related to the good overall adaptation of childhood cancer survivors reported in the literature, e.g., [3,4,5,6]. In our study sample, only six respondents reported significant PTSD symptomatology. In the case of generally low levels of PTSS/PTSD in the study sample, it would be difficult to document gender differences. A similar explanation may be relevant also for the non-applicability of variables reflecting the style of parenting in the prediction of PTSS.

In the prediction of PTSS and PTG, medical variables such as the severity of late effects and the time from treatment completion do not play a significant role. The severity of late effects is determined by physicians using CTCAE (Common Terminology Criteria for Adverse Events) and it does not reflect the subjective experience of adolescents. Subjective perception and assessment of late effects could be to some extent reflected in the MMQL scale of psychological functioning (negative emotionality), which is statistically significant in predicting PTSS, and which includes such items as “how often do you feel tired, how often do you feel strong and healthy, how often do you have concerns about your health”. The importance of the subjective assessment of factors associated with the disease and its treatment for the development of PTSS/PTSD have been corroborated by review studies by Taïeb et al. [2] and Bruce [9].

### Limitations and Further Directions

The findings of this study should be interpreted with awareness of several limitations. We assumed that the oncological disease poses a traumatic experience and in determining their PTG, respondents in this research were directly referred to the oncological disease as a traumatic experience to which the items of the BFSC questionnaire should refer. It turns out, however, that this assumption may not be applicable for all childhood cancer survivors. Within the time horizon of five years from the disease diagnosis, an event related to cancer and its treatment is reported as the most traumatic event by only about a half of the children and adolescents, and after a longer period of time by fewer than a quarter [42].

Analyses presented in this study were performed on a relatively small number of respondents. Moreover, two subscales of the “Parent-Child interaction” scale used for assessing various aspects of parenting showed relatively low levels of reliability (inconsistency of parenting, parental involvement” subscales). Twenty-three respondents were excluded from the longitudinal analyses because of various difficulties in completing the entire array of required methods (for details please see Sample and Procedure section). The exclusion of these respondents might have influenced our results.

The sample size limits the generalizability of this study. However, data collection for the QOLOP project continues and the results will be tested again by replicate analyses, and possibly supplemented with the analyses of other factors (e.g., type of cancer).

## 4. Materials and Methods

Methods monitoring PTSS and PTG were used only at T2 measurement, other reported methods were used in both T1 and T2 measurements.

PTSS were assessed using the University of California at Los Angeles Posttraumatic Stress Disorder Index, UCLA_PTSD [43]. The method includes 22 items ascertaining the frequency of PTSS in the past month on a five-point scale (none–most). Items that in the original general wording referred to “what happened” were reformulated to relate directly to the suffered disease. For example the item “How much of the time during the past month did you have dreams about what happened, or other bad dreams” has been reworded to “dreams about the disease or other bad dreams.” The reliability of the Czech translation of UCLA_PTSD was good (Cronbach’s alpha = 0.896).

PTG in terms of benefit finding was determined by Benefit Finding Scale for Children, BFSC; [13,14], which consists of 10 items related to cancer experience evaluated on the five-point scale (not at all true–very true). The reliability of the Czech translation of BFSC was good (Cronbach’s alpha = 0.897).

Negative emotionality was investigated using a scale of psychological functioning from The Minneapolis-Manchester Quality of Life method; MMQL [44,45]. The scale includes nine items focused on the intensity of feelings of sadness, loneliness, feelings of inferiority and other negative emotions, with the available answers being “always, usually, sometimes, occasionally, never”. The total score was expressed as the mean response to the items, with higher scores indicating higher levels of negative emotionality. The reliability of the Czech translation of this scale in Czech translation was satisfactory (Cronbach’s alpha = 0.716 at T1; Cronbach’s alpha = 0.711 at T2).

Subjective view of adolescents on various aspects of parenting was investigated using the Parent-Child Interactions Scale from the Social and Health Assessment questionnaire; SAHA [46]. Specifically, it includes the scale of inconsistency of parenting (Cronbach’s alpha = 0.471), the scale of parental involvement (Cronbach’s alpha = 0.449), control (Cronbach’s alpha = 0.638), and warmth (Cronbach’s alpha = 0.635). Children evaluate parents’ behavior on a four-point scale (never–often). Level of individual parenting aspects is expressed as the mean score.

Depressive symptomatology was examined through Children’s Depression Inventory, CDI [47]. The method includes 27 items ascertaining the incidence/severity of depressive symptoms. It consists of five subscales: negative mood, interpersonal problems, negative self-esteem, ineffectiveness and anhedonia. For purposes of analysis in this study, we used only the total score. Further information about the Czech version of this method can be found in Preiss [48]. The reliability of CDI was good (Cronbach’s alpha = 0.845).

Self-esteem was assessed through Rosenberg Self-Esteem Scale; RSES [49], which consists of 10 items designed to measure global self-assessment using a four-point scale for responses ascertaining the degree of acceptance of individual items (false–true). The RSES was administered only to respondents aged 14 and over. Further information about the Czech version of this method can be found in Blatný and Osecká [50]. The reliability of RSES was good (Cronbach’s alpha = 0.842).

The severity of late effects was evaluated by a physician according to CTCAE (Common Terminology Criteria for Adverse Events v3.0) so that the most serious of the occurring late effects was crucial to the resulting degree of severity.

## 5. Conclusions

To conclude, it can be claimed that PTG and PTSS are more affected by factors of parenting and the emotionality of childhood cancer survivors than by objective medical data. The results of this study suggest that warm parent-child interactions may facilitate the process of finding benefits in traumatic experience and imply the importance of including parents/caregivers in the clinical care of childhood cancer survivors.

## Figures and Tables

**Table 1:** Sample characteristics.

Characteristic		Whole Sample	Sample for Longitudinal Analyses
		(Completed T1 and T2)	(Without Shortened Version at T1)
		n = 120	n = 97
Males		59 (49.2%)	47 (48.5%)
Females		61 (50.8%)	50 (51.5%)
Dg.	CNS	11 (9.2%)	11 (11.3%)
	Leukemia	56 (46.7%	42 (43.3%)
	Extracranial solid tumor	53 (44.2%)	44 (45.4%)
Late effects	No	64 (53.3%)	49 (50.5%)
	Mild	36 (30.0%)	30 (30.9%)
	Moderate	12 (10.0%)	11 (11.3%)
	Severe	8 (6.7%)	7 (7.2%)

CNS: Central Nervous System; Dg.: Diagnosis.

**Table 2:** Results of correlations of posttraumatic stress and posttraumatic growth with selected variables (n = 116).

Variable	PTSS	Self-Esteem ^1^	Negative Emotionality	Depressive Symptoms	Severity of Late Effects	Age at Diagnosis
PTSS	—	−0.614 **	0.655 **	0.564 **	0.042	0.086
PTG	0.050	0.290 *	0.132	−0.320 **	0.045	0.283 **

^1^ Rosenberg Self-Esteem Scale was administrated only to children over 14 years of age and therefore this analysis was performed on a lower number of respondents (n = 78). * *p* ≤ 0.05; ** *p* ≤ 0.01.

**Table 3:** Results of regression analysis with considered predictors (*n* = 94).

Method	Variable	PTG (BFSC)			PTSS (UCLA_PTSD)		
		Beta	Sig	Corr	Beta	Sig	Corr
	Gender	−0.415	0.021	−0.247	−0.144	0.191	−0.163
	Age	0.252	0.013	0.161	0.034	0.739	0.125
Severity of late effects		−0.033	0.745	0.063	0.046	0.658	0.030
SAHA	Inconsistency of parenting	0.099	0.333	−0.053	0.043	0.685	0.153
	Parental involvement	−0.124	0.314	0.174	−0.156	0.221	−0.208
	Parental control	−0.086	0.417	−0.012	0.057	0.609	−0.060
	Parental warmth	0.527	0.000	0.400	−0.147	0.242	−0.253
MMQL	Negative emotionality	0.006	0.950	0.069	0.313	0.003	0.372
	Time off-treatment at T2	−0.098	0.318	−0.069	0.101	0.323	0.163
		R2 = 0.291, sig = 0.000			R2 = 0.234, sig = 0.006		

Note. In case of gender (1 = male, 0 = female) unstandardized coefficient is stated.
